# Quality-of-life in insect venom allergy: validation of the Turkish version of the “Vespid Allergy Quality of Life Questionnaire” (VQLQ-T)

**DOI:** 10.1186/s40064-016-2246-x

**Published:** 2016-05-10

**Authors:** Betül Ayşe Sin, Derya Öztuna, Aslı Gelincik, Feridun Gürlek, Abdullah Baysan, Aytül Zerrin Sin, Ömür Aydın, Zeynep Mısırlıgil

**Affiliations:** Division of Immunology and Allergy, Department of Pulmonary Diseases, School of Medicine, Ankara University, Ankara, Turkey; Department of Biostatistics, School of Medicine, Ankara University, Ankara, Turkey; Division of Immunology and Allergy, Department of Internal Medicine, Istanbul University, Istanbul, Turkey; Division of Immunology and Allergy, Department of Internal Medicine, Ege University, Izmir, Turkey; Division of Immunology and Allergy, Department of Internal Medicine, School of Medicine, Gülhane Military Hospital, Ankara, Turkey

**Keywords:** Venom allergy, Honey bee, Vespid, Quality of Life, Questionnaire

## Abstract

**Purpose:**

“Vespid Allergy Quality of Life Questionnaire (VQLQ)” has been used to assess psychological burden of disease. The aim of this study was to evaluate validity, reliability and responsiveness to interventions of the Turkish version.

**Methods:**

The Turkish language Questionnaire (VQLQ-T) was administered to 81 patients with bee allergy and 65 patients with vespid allergy from different groups to achieve cross-sectional validation. To establish longitudinal validity, the questionnaire was administered to 36 patients treated with venom immunotherapy.

**Results:**

The cross-sectional validation in patients with vespid venom allergy showed a correlation coefficient of 0.97 (Cronbach α). Spearman’s correlation coefficient of the pretreatment VQLQ-T score with Expectation of Outcome (EoO) questionnaire score was 0.55 (p < 0.001). After treatment, correlation between VQLQ-T score and EoO score was 0.64 (p = 0.003) in these patients. The cross-sectional instrument validation for non-beekeepers with bee venom allergy yielded a correlation coefficient of 0.96 (Cronbach α). Spearman’s correlation coefficient between pretreatment VQLQ-T score and EoO score was 0.47 (p < 0.001) and after treatment, correlation between VQLQ-T score and EoO score was 0.78 (p = 0.008) in these patients. These findings indicate cross-sectional validity of VQLQ-T. In the longitudinal validation, there was a positive correlation between EoO and VQLQ-T with a correlation coefficient of 0.562 (p < 0.001). While mean (±SD) VQLQ-T score was 5.27 (±1.29) in pretreatment, it was 2.78 (±1.01) after treatment (p < 0.001). The correlation between the mean change in VQLQ-T score and the mean change in EoO score was 0.42 (p = 0.011).

**Conclusions:**

The Turkish version of VQLQ-T enables measurement of Quality of Life (QoL) in patients with either vespid or bee venom allergy. Furthermore, responsiveness of this instrument demonstrates the questionnaire’s ability to detect changes over time.

## Background

In recent years, Quality of Life (QoL) issues have become an interesting topic in many diseases. Health-related quality-of-life (HRQoL) questionnaires are increasingly used in both research and patient care as they enable the evaluation of health status and treatment outcomes relevant to the patient (Oude Elberink 2006). Several studies have shown that allergic diseases are frequently associated with a diminished QoL, which in turn also affects healthcare costs (Franzese and Burkhalter 2010; Teufel et al. 2007). However, little is known about the Quality of Life of patients who are allergic to insect venom. Disease-specific questionnaires are preferred for specific allergic diseases and thought to be more sensitive than the generic ones (Baiardini et al. 2003). Hymenoptera venom allergy is one of the most important causes of life-threatening allergic events. Different from the allergic diseases with ongoing symptoms, patients suffering from systemic sting reactions occasionally experience symptoms due to the unexpected venom exposure. However, this special group of patients may still be found to have an impaired QoL due to venom allergy as a result of their efforts to prevent accidental exposures (Oude Elberink et al. 2002a).

The intensity of systemic reaction to insect stings ranges between mild cutaneous and severe allergic manifestations, with anaphylactic shock even leading to death in some cases. Due to the characteristic nature of the disease, the impact of insect venom allergy on HRQoL may be associated with several social restrictions and limitation of daily activities for subjects. In recent years, although QoL and the psychological burden of disease in special patient groups such as food and drug allergy are increasingly important topics in medical care and clinical research, there is limited knowledge about the QoL of patients with venom allergy (Oude Elberink 2006; Teufel et al. 2007; Gowland 2002; Antolin-Amerigo et al. 2015; Baiardini et al. 2015). This may be due to the methodological difficulties in developing a questionnaire survey. Measurement of HRQoL in patients with venom allergy was first proposed by Confino-Cohen et al. (1999). They used a non-standardized questionnaire, and showed that approximately one-third of patients receiving venom immunotherapy (VIT) perceived a moderate to severe impairment in their QoL and manifested symptoms of emotional distress despite ongoing therapy (Confino-Cohen et al. 1999). Later on in 2002, Oude Elberink et al. developed and validated the first disease-specific instrument called ‘Vespid Allergy Quality of Life Questionnaire’ (VQLQ) to measure HRQoL in patients with anaphylaxis due to *Vespula* venom (Oude Elberink et al. 2002a). To date, this tool has not been used to assess the validity and reliability of VQLQ scores in patients with bee venom allergy.

The prevalence of systemic reactions due to Hymenoptera stings in the general adult population was reported as between 1.2 and 4.3 % in Turkey, which was in line with representative studies from Europe and US (Gelincik et al. 2015; Tankersley and Ledford 2015). However, the Quality of Life scores of patients with venom allergy and especially in those receiving VIT has not yet been evaluated in Turkey. Therefore, in the present study we aimed to assess the validity and reliability of the Turkish version of the VQLQ both cross-sectionally and longitudinally in adults with venom allergy. The secondary objectives were to evaluate the VQLQ use in patients with bee venom allergy and responsiveness of the tool to intervention in patients treated with VIT.

## Methods

### Patient selection

Four centers from Turkey participated in the study between the years 2010 and 2013. A total of 189 adult patients [103 M, 86 F mean age: 41.17 ± 12.26 years, median (min–max): 42 (17–70)] who had a history of systemic sting reactions (SSRs) were screened for the study in the Allergy Outpatient Departments of these centers. Venom allergy to *Vespula* and/or *Apis* species was confirmed by positive skin test response and/or elevated titers of serum specific IgE antibodies (Bilo et al. 2005). Severity of the reactions ranged from mild to severe, which was graded according to the method of Mueller (Mueller 1966).

### Specific IgE measurements/sensitization

Skin tests were performed with standardized pure venom extracts of *Vespula vulgaris* and *Apis mellifera* (ALK-Abello, Madrid, Spain). Skin prick tests were applied by using 100 and 300 μg/ml concentrations. Histamine dihydrochloride (10 mg/ml) and glycerol diluent were used as positive and negative controls, respectively. A wheal diameter with erythema of 3 mm or greater than histamine, was accepted as a positive reaction. If the prick test result was negative, intradermal tests with the standard extracts of the same venom allergens (ALK-Abello, Spain) were performed in tenfold increasing concentrations from 0.0001 µg/ml to a maximum of 1 µg/ml of 0.02 ml of venom. Intradermal test response with a histamine equivalent wheal size of at least 5 mm at a concentration of less than 1 μg/ml was considered to be a positive reaction. Histamine and physiological saline with 0.4 % phenol were used as positive and negative controls, respectively. The skin test results were read after 15 min. Patients were diagnosed as sensitized to *Vespula vulgaris* and/or *Apis mellifera* venoms. In all patients, specific IgE antibodies to *Vespula vulgaris* and *Apis mellifera* venoms were determined by using a florescence-immunoassay (Pharmacia CAP, Uppsala, Sweden). Antibody values of 0.35 kU/L or more were classified in the range of class 1 and class 6. Specific IgE result of at least class 2 was considered positive.

### Instruments

We used the Turkish version of the Vespid Allergy Quality of Life Questionnaire (VQLQ-T) which was previously developed and validated in 2002 by Oude Elberink JNG with her permission (Oude Elberink et al. 2002a).

### Translation and backtranslation of the English version

In order to use the Vespid Allergy Quality of Life Questionnaire (VQLQ) in Turkish, the VQLQ was first forward translated into Turkish by a person who speaks both English and Turkish. Then, it was independently backward translated from Turkish into English by another person in order to evaluate the accuracy of the translation. The translation was carried out without any changes by two academic staff at the Department of Western Languages and Literatures in the Faculty of Languages, History and Geography at the Ankara University (Zeynep Atayurt-Fenge and Devrim Kılıcer). In this way, the final version of the questionnaire-The Turkish Language Questionnaire (VQLQ-T) was agreed upon by the authors of the original version, and the validation process was begun.

The original questionnaire consisted of 14 questions with 7 response options giving a score of 1–7, with lower scores representing higher QoL. In addition, there were two questions asking the patient about the likelihood of a severe allergic reaction or even death after being re-stung by a vespid or bee. The answers to these questions were used for validation of the previous 14 questions. The first six questions of this survey were related to anxiety symptoms in various forms (agitation) and avoidance and fear behavior (running away, avoiding certain places, need to have control). The other eight questions were related to stress due to increased vigilance. These are posed for general situations (e.g. being outdoors) as well as specific everyday situations (at work, while eating outside, gardening, being in nature or on vacation). Most of the questions were related to the emotional aspects of the patient’s life, although questions related to work, leisure, and outside activities were also included (Oude Elberink et al. 2002a).

The VQLQ-T, consisting of 14 items along with the Expectation of Outcome (EoO) questions (2 items), was administered to patients to achieve cross-sectional validation of the Turkish version. The EoO questionnaire was required to use as an external reference point for the validation. This had previously been used in the validation process of the original version, and consisted of 2 questions related to the risk perceived by patients of experiencing a severe allergic reaction or dying as a result of a new hymenoptera sting (Oude Elberink et al. 2002a).

In order to evaluate the validity of VQLQ-T for patients with bee venom allergy, the questionnaire was also applied to patients to identify issues in their daily lives affected by their allergy to honey bees.

#### Longitudinal validation of the questionnaire

A total of 36 patients [19 M/17 F, mean age: 46.25 ± 9.32 years, median (min–max): 46.5 (28–69)] were included in the longitudinal validation. The VQLQ-T and the EoO Questionnaires were applied to patients prior to the decision of treatment modality. Consequently, in 27 patients who were sensitized to only bee or vespid venom and in 9 patients who were both sensitized to vespid and bee venom, the measures were administered before VIT. After 3 years of treatment, the set of measures was re-administered to establish longitudinal validity in these patients. The longitudinal item-based validation were examined over 36 patients (overall) and 27 patients (only with bee or vespid venom allergy).

#### Cross-sectional validation of the questionnaire

Patients referred to the out-patient allergy departments because of a systemic allergic reaction after a vespid and/or honey bee sting were asked to complete the VQLQ-T along with the EoO questions. For the cross-sectional validation, we included patients aged over 17 years who were confirmed to be sensitized with the culprit insect after a systemic allergic reaction to venom from *Vespula* or *Apis* species. This questionnaire (VQLQ-T) was administered to 81 patients with bee venom allergy and 65 patients with vespid venom allergy. Forty-three patients demonstrated double sensitization. Those patients who were sensitive to both venoms were excluded from the analysis in order to avoid the misinterpretation of the data of patients with only bee or vespid venom allergy.

Patients were separated into four different groups depending on their current status. The first group (1) consisted of patients who were recently diagnosed (n = 56), the second group (2) had patients who have recently started VIT but have not yet completed up-dosing (n = 64). Patients in the maintenance phase of VIT formed the third group (3) (n = 48) and finally patients who had completed the VIT were in the fourth group (4) (n = 21) (Table 1). We applied VQLQ-T questionnaire to these groups at the same time point.

**Table 1:** Characteristics of patients with vespid or bee venom allergy

	*Apis*	*Vespula*
Number	81	65
Age (years)*	42.33 ± 12.58	39.2 ± 13.06
	[46 (18–70)]	[38 (17–70)]
Gender		
Female, no (%)	34 (42 %)	33 (51 %)
Male, no (%)	47 (58 %)	32 (49 %)
Severity of systemic reactions (%)		
Grade I	9 (11.1)	5 (7.7)
Grade II	21 (26)	25 (38.5)
Grade III	24 (29.6)	15 (23)
Grade IV	27 (33.3)	20 (30.8)
Patient groups for cross-sectional validation		
New diagnosis	18 (22.2)	23(35.4)
Recently started (up-dosing) VIT	30 (37)	18 (27.7)
Receiving on maintenance VIT	22 (27.2)	16 (24.6)
Stopped VIT	11 (13.6)	8 (12.3)

For the variables with *, cells represent mean ± standard deviation [median (min–max)], for the others, cells represent frequency (percent). *VIT* venom immunotherapy

All of the patients, who participated in the study, gave their written informed consent. This study was approved by the Medical Ethics Committee of the Ankara University, Ankara, Turkey. All procedures performed in this study were in accordance with the ethical standards of the institutional research committee and with the 1964 Helsinki declaration and its later amendments or comparable ethical standards.

### Statistical analysis

Statistical analyses were performed using SPSS 11.5. Mean ± standard deviation [median (minimum–maximum)] for metric variables, and frequency (percent) for categorical variables were given as descriptive statistics. In order to compare independent groups in terms of metric variables, the Mann–Whitney U test was used for two groups, while the Kruskal–Wallis variance analysis was used when more than two groups were involved. Paired samples t test was performed to compare the two dependent groups for metric variables. The Pearson/Spearman correlation coefficient was calculated to determine the association between variables, and p < 0.05 was considered statistically significant.

### Scale (VQLQ-T) reliability

Reliability was tested by Cronbach’s alpha coefficient (Cronbach 1951). Usually a reliability of 0.70 is required for analysis at group level, and values of 0.85 and higher for individual use (Streiner and Norman 1995).

### Scale (VQLQ–T) validity

Cross-sectional validity (as internally) of the VQLQ-T was tested with item-scale correlations. Also, the correlation between the VQLQ-T and the EoO (for pre treatment and after treatment) was given at both the item and scale levels in terms of external validation. For longitudinal validation (responsiveness), the paired t test was used for internal responsiveness, correlation between the change score of the VQLQ-T and the EoO was calculated for external responsiveness.

## Results

### Patient population

Patients filled out the questionnaire in approximately 10–15 min without requiring any assistance. The distribution of venom sensitizations were honey bee in 81 subjects (42.8 %), vespid in 65 patients (34.5 %), and a double sensitization both to honey bee and vespid was observed in 43 patients (22.7 %). There was no difference in the VQLQ-T scores in terms of the type of insect venom responsible for sensitization (Kruskal–Wallis Chi square test statistic = 0.669; p = 0.880).

All patients had experienced a systemic reaction after a vespid and/or a bee sting. The severity of reactions was graded according to the method of Mueller (1): Grade I: n = 21 (11.1 %), Grade II: n = 56 (29.6 %), Grade III: n = 55 (29.1 %), Grade IV: n = 57 (30.2 %).

A positive correlation between the degree of severity of the systemic reaction and the VQLQ-T scores (correlation coefficient r = 0.041) was found, but it did not reach statistical significance (p = 0.578, > 0.05). Furthermore, the degree of systemic reaction severity was significantly correlated with the skin test reactivity to vespula venom (r = 0.408, p = 0.025) and wheal diameter of histamine (r = 0.317, p < 0.001).

The mean duration of VIT was 4.5 ± 1.5 years for *Apis mellifera*, and it was 5.0 ± 0.1 years for *Vespula vulgaris* venom. There was a statistically significant difference in VQLQ scores between the recently started VIT group (mean ± SD: 5.03 ± 1.45 median: 5.4 min–max: 1.38–7.00) and the group who had completed VIT (mean ± SD: 4.35 ± 1.29 median: 4.3 min–max: 1.93–6.31) (Mann–Whitney U test statistic = 812,500; p = 0.028). The mean VQLQ score was significantly lower in patients whose VIT had stopped. Furthermore, the VQLQ-T scores were inversely correlated with the duration of VIT (correlation coefficient: −0.267, p = 0.378).

### Longitudinal instrument validation (responsiveness)

Longitudinal validity was studied in 36 patients as previously mentioned. After 3 years, the questionnaire was re-administered to patients treated with VIT. There was a positive correlation between the pretreatment EoO and the VQLQ-T with a correlation coefficient of 0.562, and this coefficient was statistically significant (p < 0.001). While the mean (±SD) VQLQ-T score was 5.27 (±1.29) in pretreatment, it was 2.78 (±1.01) after treatment (p < 0.001). This finding supports the internal responsiveness of the VQLQ-T. The correlation between the mean change in the VQLQ-T score (mean ± SD: 2.48 ± 1.23) and the mean change in the EoO score (mean ± SD: 3.15 ± 1.8) was 0.42 (p = 0.011) indicating the external longitudinal construct validity of the VQLQ-T. The results of longitudinal item-based validation over 36 patients (overall) and 27 patients (only with bee or vespid venom allergy) are given in Tables 2 and 3, respectively.

**Table 2:** Longitudinal item-based validation of the Turkish VQLQ and EoO (n = 36)

	Before	After	Difference	p value
*VQLQ*				
VQLQ-1	5.58 ± 1.51 [6 (1–7)]	3.05 ± 1.68 [3 (1–7)]	2.53 ± 1.59	<0.001
VQLQ-2	5.11 ± 1.66 [6 (2–7)]	2.61 ± 1.59 [3 (1–7)]	2.5 ± 1.73	<0.001
VQLQ-3	5.86 ± 1.41 [6 (2–7)]	3.52 ± 1.61 [3 (1–7)]	2.33 ± 1.62	<0.001
VQLQ-4	5.08 ± 1.82 [6 (1–7)]	2.72 ± 1.52 [3 (1–7)]	2.36 ± 1.74	<0.001
VQLQ-5	5.14 ± 1.79 [6 (2–7)]	2.97 ± 1.66 [3 (1–7)]	2.17 ± 1.95	<0.001
VQLQ-6	4.94 ± 2.12 [6 (1–7)]	2.88 ± 1.51 [3 (1–7)]	2.06 ± 1.80	<0.001
VQLQ-7	4.65 ± 2.08 [5 (1–7)]	2.22 ± 1.37 [2 (1–7)]	2.43 ± 1.93	<0.001
VQLQ-8	5.37 ± 1.66 [6 (1–7)]	2.74 ± 1.31 [3 (1–5)]	2.63 ± 1.77	<0.001
VQLQ-9	5.63 ± 1.23 [6 (3–7)]	2.86 ± 1.49 [3 (1–7)]	2.77 ± 1.68	<0.001
VQLQ-10	5.18 ± 1.65 [6 (1–7)]	2.77 ± 1.47 [3 (1–7)]	2.41 ± 1.68	<0.001
VQLQ-11	5.22 ± 1.62 [5 (1–7)]	2.56 ± 1.08 [3 (1–5)]	2.65 ± 1.58	<0.001
VQLQ-12	5.55 ± 1.63 [6 (2–7)]	2.95 ± 1.50 [2.5 (1–6)]	2.6 ± 1.47	<0.001
VQLQ-13	5.09 ± 1.59 [6 (2–7)]	2.75 ± 1.34 [3 (1–6)]	2.34 ± 1.56	<0.001
VQLQ-14	5.74 ± 1.37 [6 (2–7)]	2.80 ± 1.32 [3 (1–6)]	2.94 ± 1.55	<0.001
*EoO*				
EoO-1	5.83 ± 1.18 [6 (2–7)]	2.58 ± 1.31 [2 (1–7)]	3.25 ± 1.78	<0.001
EoO-2	5.97 ± 1.14 [6 (3–7)]	2.73 ± 1.42 [3 (1–7)]	3.24 ± 1.94	<0.001

VQLQ 1–14 (Vespid allergy quality of life questionnaire), *EoO* Expectation of Outcome questionnaire (questions 1 and 2) (independent measure)

**Table 3:** Longitudinal item-based validation of the Turkish VQLQ and EoO for vespid and bee venom allergic patients (n = 27)^a^

	Vespid venom allergic patients (n = 11)					Bee venom allergic patients (n = 16)				
	Before	After	Difference	Correlation	p value	Before	After	Difference	Correlation	p value
*VQLQ*										
1	5.27 ± 2 [6 (1–7)]	2.09 ± 1.64 [1 (1–6)]	3.18 ± 1.83	0.509	0.005	5.69 ± 1.45 [6 (2–7)]	3.31 ± 1.54 [4 (1–6)]	2.38 ± 1.36	0.586*	0.001
2	5.55 ± 1.37 [6 (3–7)]	2.64 ± 1.86 [2 (1–6)]	2.91 ± 1.70	0.479	0.005	4.88 ± 1.86 [5.5 (2–7)]	2.44 ± 1.36 [2.5 (1–6)]	2.44 ± 1.67	0.496*	0.001
3	5.91 ± 0.83 [6 (4–7)]	4 ± 2.05 [3 (1–7)]	1.91 ± 1.76	0.528	0.017	5.44 ± 1.86 [6.5 (2–7)]]	3.19 ± 1.52 [3 (1–7)]	2.25 ± 1.73	0.489*	0.002
4	4.73 ± 1.95 [6 (2–7)]	2.09 ± 0.94 [2 (1–3)]	2.64 ± 2.01	0.177	0.011	5.19 ± 1.91 [6 (1–7)]	2.94 ± 1.65 [3 (1–7)]	2.25 ± 1.73	0.534*	0.002
5	5.09 ± 1.97 [6 (2–7)]	2.64 ± 1.75 [2 (1–6)]	2.45 ± 2.21	0.301	0.011	4.88 ± 1.82 [5.5 (2–7)]	2.75 ± 1.65 [2.5 (1–6)]	2.13 ± 2.13	0.255	0.004
6	4.27 ± 2.37 [4 (1–7)]	2.82 ± 1.83 [2 (1–6)]	1.45 ± 1.57	0.749**	0.026	5 ± 2.33 [6 (1–7)]	2.80 ± 1.32 [3 (1–5)]	2.20 ± 1.78	0.650**	0.003
7	5.09 ± 2.12 [6 (1–7)]	2 ± 1.34 [1 (1–5)]	3.09 ± 2.07	0.352	0.007	3.93 ± 2.12 [4 (1–7)]	1.80 ± 1.01 [1 (1–3)]	2.13 ± 1.92	0.425	0.003
8	5.45 ± 2.02 [6 (1–7)]	2.18 ± 1.40 [2 (1–5)]	3.27 ± 2.05	0.321	0.007	5.40 ± 1.72 [6 (2–7)]	2.87 ± 1.19 [3 (1–5)]	2.53 ± 1.73	0.342	0.001
9	5.64 ± 1.36 [6 (3–7)]	2.36 ± 1.63 [2 (1–6)]	3.27 ± 2.00	0.111	0.007	5.67 ± 1.18 [6 (3–7)]	2.93 ± 1.28 [3 (1–6)]	2.73 ± 1.49	0.269	0.001
10	6 ± 1.55 [6.5 (3–7)]	2.67 ± 1.63 [2.5 (1–5)]	3.33 ± 2.16	0.079	0.043	4.60 ± 1.90 [5 (1–7)]	2.30 ± 1.06 [2.5 (1–4)]	2.30 ± 1.34	0.730*	0.007
11	5.5 ± 1.20 [6 (3–7)]	2.25 ± 1.04 [3 (1–3)]	3.25 ± 1.67	−0.115	0.016	4.89 ± 2.03 [5 (1–7)]	2.78 ± 1.09 [3 (1–5)]	2.11 ± 1.27	0.834**	0.010
12	5.2 ± 1.92 [6 (2–7)]	2.6 ± 1.82 [2 (1–5)]	2.60 ± 1.52	0.673	0.042	5.9 ± 1.6 [6.5 (3–7)]	3 ± 1.56 [2.5 (1–6)]	2.90 ± 1.37	0.624*	0.005
13	5 ± 1.58 [6 (3–7)]	2.11 ± 1.05 [3 (1–3)]	2.89 ± 1.90	0	0.017	5 ± 1.69 [6 (2–7)]	3.13 ± 1.64 [3 (1–6)]	1.87 ± 1.46	0.618*	0.002
14	5.64 ± 1.69 [6 (2–7)]	2.09 ± 1.22 [2 (1–5)]	3.55 ± 2.07	0.018	0.007	5.73 ± 1.44 [6 (3–7)]	3.07 ± 1.28 [3 (1–5)]	2.67 ± 1.35	0.515*	0.001
*EoO*										
1	6.09 ± 0.94 [6 (4–7)]	2.09 ± 0.7 [2 (1–3)]	4 ± 1.18	−0.014	0.003	−5.38 ± 1.75 [6 (2–7)]	2.56 ± 1.03 [2 (1–5)]	2.81 ± 2.01	0.023	0.001
2	6.55 ± 0.52 [7 (6–7)]	2.18 ± 0.87 [2 (1–3)]	4.36 ± 0.67	0.637*	0.003	5.38 ± 1.54 [6 (3–7)]	2.56 ± 0.96 [2 (1–5)]	2.81 ± 1.87	0.062	0.001

* p < 0.05; ** p < 0.01; *** p < 0.001

^a^Both vespid and bee venom allergic patients (n = 9) were excluded from the analyses

### Cross-sectional validation of individual items in vespid venom allergy (n = 65)

For the cross-sectional validity; characteristics of patients with vespid and bee venom allergy depending on different groups are shown in Table 1.

The VQLQ-T consists of 14 items with inter-item correlation coefficients ranging between 0.53 and 0.97. The Cronbach α of the 14 items was 0.97. The Spearman’s correlation coefficients between the individual items, the mean of all items and the EoO questionnaire are given in Table 4. The Spearman’s correlation coefficient between the pretreatment VQLQ-T score (mean ± SD: 4.7 ± 1.58) and EoO questionnaire score (mean ± SD: 5.23 ± 1.41) was 0.55 (p < 0.001). After treatment, the correlation between the VQLQ score (mean ± SD: 2.81 ± 1.07) and the EoO score (mean ± SD: 2.8 ± 1.53) was 0.64 (p < 0.003). These findings indicate the cross-sectional validity of the VQLQ-T.

**Table 4:** External validity of the Turkish VQLQ for vespid allergy (n = 65)

Number of items	Expectation of outcome		
	Q1	Q2	Mean of Q1 and Q2
Q1	0.466***	0.443***	0.494***
Q2	0.477***	0.494***	0.522***
Q3	0.469***	0.332**	0.435***
Q4	0.291*	0.273*	0.301*
Q5	0.455***	0.309*	0.389**
Q6	0.515***	0.240*	0.408**
Q7	0.394**	0.520***	0.501***
Q8	0.423**	0.372**	0.415**
Q9	0.567***	0.434***	0.535***
Q10	0.370*	0.351*	0.412**
Q11	0.567***	0.546***	0.611***
Q12	0.459*	0.214*	0.350*
Q13	0.428**	0.453**	0.480***
Q14	0.566***	0.354**	0.501***
Mean (Q1–14)	0.536***	0.469***	0.549***

*Q* question

* p < 0.05; ** p < 0.01; *** p < 0.001

### Longitudinal instrument validation in vespid venom allergy (n = 11)

The pretreatment mean (±SD) VQLQ-T score was 5.27 (±1.41), while it was 2.5 (±1.08) after treatment (p < 0.001). This finding supports the internal responsiveness of the VQLQ-T. The correlation between the mean change in the VQLQ-T score (mean ± SD: 2.41 ± 1.0) and the mean change in the EoO score (mean ± SD: 2.50 ± 2.21) was 0.67 (p = 0.050), indicating the external longitudinal construct validity of the VQLQ-T.

### Cross-sectional validation of individual items in bee venom allergy (n = 81)

The VQLQ-T revealed correlation coefficients which were ranging between 0.48 and 0.90 for patients with bee venom allergy. The cross-sectional instrument validation yielded a correlation coefficient of 0.96 (Cronbach α). The Spearman correlation coefficients between the individual items and the mean of all items and the EoO questionnaire are shown in Table 5. The Spearman’s correlation coefficient between the pretreatment VQLQ-T score (mean ± SD: 4.9 ± 1.45) and the EoO score (mean ± SD: 5.19 ± 1.39) was 0.49 (p < 0.001). After treatment, the correlation between the VQLQ-T score (mean ± SD: 2.76 ± 0.98) and the EoO score (mean ± SD: 2.56 ± 0.91) was 0.75 (p = 0.001). These findings indicate cross-sectional validity of the VQLQ-T for bee venom allergy.

**Table 5:** External validity of the Turkish VQLQ for honey bee allergy (n = 81)

Number of items	Expectation of Outcome		
	Q1	Q2	Mean of Q1 and Q2
Q1	0.506***	0.328**	0.437***
Q2	0.404***	0.260*	0.334**
Q3	0.503***	0.323**	0.423***
Q4	0.266*	0.247*	0.276*
Q5	0.357**	0.325**	0.362**
Q6	0.318**	0.236*	0.277*
Q7	0.471***	0.387***	0.447***
Q8	0.534***	0.412***	0.495***
Q9	0.508***	0.355**	0.450***
Q10	0.392**	0.289*	0.331*
Q11	0.340*	0.242*	0.321*
Q12	0.473***	0.409**	0.447**
Q13	0.369**	0.338**	0.365**
Q14	0.559***	0.377**	0.481***
Mean (Q1–14)	0.533***	0.414***	0.490***

*Q* question

* p < 0.05; ** p < 0.01; *** p < 0.001

### Longitudinal instrument validation in bee venom allergy (n = 16)

While the mean (±SD) VQLQ score was 5.07 (±1.36) in pretreatment, it was 2.76 (±0.98) after treatment (p < 0.001). This finding supports the internal responsiveness of the VQLQ-T. The correlation between the mean change in the VQLQ-T score (mean ± SD: 2.32 ± 1.13) and the mean change in the EoO score (mean ± SD: 2.81 ± 1.83) was 0.55 (p = 0.029) indicating the external longitudinal construct validity of the VQLQ-T for bee venom allergy.

Among patients with bee venom allergy, there were 28 (34.6 %) subjects who were doing beekeeping, while 53 (65.4 %) patients were non-beekeepers. When we analyzed their VQLQ-T scores, there was a statistically significant difference between beekeepers and non-beekeepers (p < 0.05). The mean VQLQ score was lower for beekeepers than non-beekeepers (4.4 ± 1.5 vs. 5.1 ± 1.3). When the beekeepers were excluded from the analysis, remaining 53 cases consisted of non-beekeepers. In this group; the correlation coefficients were ranging between 0.55 and 0.94. The Cronbach α of the 14 items was 0.96 in the cross-sectional validation of individual items. The Spearman’s correlation coefficient between pretreatment VQLQ-T score (mean ± SD: 5.12 ± 1.34) and EoO questionnaire (mean ± SD: 5.06 ± 1.26) was 0.47 (p = 0.001). After treatment, correlation between the VQLQ-T score (mean ± SD: 2.8 ± 0.74) and the EoO score (mean ± SD: 2.65 ± 1.11) was 0.78 (p = 0.008). These findings indicate cross-sectional validity of the VQLQ-T. Longitudinal instrument validation in non-beekeepers demonstrated that the mean (±SD) VQLQ-T score was 4.84 (±1.33) in pretreatment whereas it was 2.8 (±0.74) after treatment (p < 0.001). This finding supports the internal responsiveness of the VQLQ-T. The correlation between the mean change in VQLQ-T score (mean ± SD: 2.04 ± 1.2) and the mean change in EoO score (mean ± SD: 2.35 ± 1.84) was 0.28 (p = 0.431). However, these findings are not enough to show the external longitudinal construct validity of VQLQ-T. The Spearman correlation coefficients between the individual items and the mean of all items and the EoO questionnaire were shown in Table 6.

**Table 6:** External validity of the Turkish VQLQ in non-beekeepers with bee venom allergy (n = 53)

	Expectation of outcome		
VQLQ items	Q1	Q2	Mean of Q1 and Q2
Q1	0.479***	0.307*	0.431**
Q2	0.316*	0.182	0.249
Q3	0.411**	0.104	0.248
Q4	0.144	0.282*	0.255
Q5	0.343*	0.290*	0.345*
Q6	0.304*	0.195	0.239
Q7	0.454**	0.473***	0.512***
Q8	0.420**	0.398**	0.448**
Q9	0.431**	0.298*	0.397**
Q10	0.342*	0.287	0.296
Q11	0.300	0.306	0.372*
Q12	0.460*	0.481**	0.509**
Q13	0.425**	0.397**	0.431**
Q14	0.491***	0.313*	0.419**
Mean (Q1–14)	0.473***	0.404**	0.465**

*Q* question

* p < 0.05; ** p < 0.01; *** p < 0.001

## Discussion

The present study showed that the Turkish version of VQLQ is a reliable and valid instrument for evaluating QoL in patients with insect venom allergy. The existing original English-language survey on vespid allergy QoL questionnaire was translated and backtranslated into Turkish language (VQLQ-T) based on current guidelines, and subsequently validated both cross-sectionally and longitudinally in either patients with vespid or bee venom allergy. Furthermore, this study was the first study which used the same instrument to measure QoL of patients with bee venom allergy.

In our study, there was a high level of internal consistency and agreement with the original version. The cross-sectional validity was established for the Turkish version by correlating responses to the items with the Expectation of Outcome questionnaire. We also showed high internal consistency by comparing the inter-item correlation by means of the Cronbach α. Also, we attribute the high reliability (internal consistency) of the Turkish language version by using the method of translation/back translation. A comparison of the Pearson’s correlation coefficients of results of the surveys of the Turkish and English samples shows a good level of agreement. Additionally, the tool appeared to be responsive to interventions including venom immunotherapy.

HRQoL is often measured as a patient-reported outcome and is incorporated into clinical trials as an outcome measurement beyond morbidity and mortality (US Food and Drug Administration (FDA)). However, the use of the validated disease specific HRQoL tools are recommended for different allergies such as drug, food or insect venom hypersensitivity (Bertine et al. 2008; Baiardini et al. 2011; Bavbek et al. 2015; Oude Elberink and Dubois 2003). After the development and validation of the first disease-specific “Vespid Allergy Quality of Life Questionnaire (VQLQ)” in 2002, the validity, reliability, and sensitivity of this questionnaire has been shown in several studies. In agreement with our findings, validation of the German (VQLQ-d) and Spanish versions for adult patients, and Polish version for adolescents were also reported in addition to Dutch and English versions (Oude Elberink et al. 2002a; Fisher et al. 2011; Cichocka-Jarosz et al. 2012; Armisen et al. 2015). Cichocka-Jarosz et al. developed a new scale for children and adolescents measuring six dimensions of HRQoL related to Hymenoptera venom allergy, and demonstrated high validity and reliability (Cichocka-Jarosz et al. 2013). This suggests that this instrument is suitable for adaptation to the different cultural settings from a HRQoL point of view. Until now, no adapted or original scale to measure the QoL in venom allergic patients has been used in Turkey. Our study is the first one that adapted the VQLQ to the Turkish population. Furthermore, the reliability and validity of Turkish version of VQLQ-T were evaluated both in patients with vespid and bee venom allergy. Consistent with results from other authors, the statistical analyses provided evidence that the Turkish version of the VQLQ met the standards for good internal consistency reliability with a Cronbach’s alpha of 0.97 in patients with vespid venom allergy. The internal consistency of VQLQ-T was very high with a Cronbach alpha of 0.97 compared to the English version of 0.88. Consistent with results from other studies, the validity of VQLQ-T was found to be satisfactory as cross-sectionally, externally and longitudinally. Thus, our findings showed that the Turkish version of VQLQ-T had satisfactory psychometric properties as a measure of QoL in patients with both vespid and bee venom allergy. In addition, the questionnaire was well accepted by the patients, who were able to fill it out quickly (approximately 15 min) and without help.

To the best of our knowledge, established VQLQ instrument have not yet been validated for bee venom allergy. VQLQ-T is the first questionnaire to include *Apis*-allergic patients in its validation process. We excluded patients who were beekeepers from the analyses, as in the first study conducted by Oude Elberink et al. (2002a). In their study, it is suggested that this instrument could be suitable for non-beekeepers who are allergic to honey bees. Since the allergy is an occupational disease in beekeepers, the approach to allergy in this group is different from the general population. As expected, beekeepers demonstrated better VQLQ scores than the non-beekeepers who were allergic to honey bees in our study. Despite this, the analysis of the internal consistency of the VQLQ-T yielded a Cronbach α of 0.96, which can be considered to be excellent, and VQLQ-T showed a significant positive correlation with the EoO score in non-beekeepers. Therefore, it should be noted that this instrument is suitable for adult patients with bee venom allergy.

Items with lower correlations relating to check certain places for stinging insects (item 6) and watch out at work (item 10) may be probably due to less frequent exposure to honey bees. However, increased fear level when a subject is stung by an insect (item 4) and in specific settings where stinging insects are likely occur (items 11 and 12), concern about vigilance correlates to sting outcome expectations to a comparable degree in vespid and bee venom allergic patients. From another point of view, items with low correlations related to gardening activities in vespid allergic patients may be due to low exposure to gardening activities in our population. On the other hand, in honey bee-venom allergic patients, correlations were found to be low relating to gardening, workplace activities and holidays. This may also be explained with rare exposure when compared to vespid allergic patients. However, correlations related to summertime activities were found to be significantly high in both vespid and honey bee allergic group.

It is well-known that venom immunotherapy (VIT) is very effective at preventing further anaphylactic reactions after insect stings. Although Confino-Cohen et al. showed that the QoL scores in venom allergic patients did not improve after VIT with their non-standardized questionnaire (Confino-Cohen et al. 1999), studies using disease-specific instrument (VQLQ) have demonstrated that VIT resulted in a significant improvement in QoL scores of patients either who have treated with VIT or after tolerated sting challenge procedure (Cichocka-Jarosz et al. 2012; Oude Elberink et al. 2002b; Fisher et al. 2013). Also, this questionnaire was previously used to compare patients receiving VIT or adrenalin auto-injectors. Oude Elberink et al. revealed that of every 3 patients treated with VIT, 2 patients experience an important improvement in their QoL after treatment (Oude Elberink et al. 2002b). In agreement with this data, we observed significantly lower VQLQ scores after VIT, consequently a high score on the VQLQ could reinforce starting VIT in patients with mild cutaneous systemic reactions (Oude Elberink et al. 2009). We believe that the use of the VQLQ-T in daily clinical practice could provide a useful tool for initial approaches to patients with venom allergy because those with a very poor QoL may need more efforts from the allergist to reassure them with regard to starting VIT (Findeis and Craig 2014).

It is already well-known that assessing the patient’s subjective point of view via disease-specific questionnaire offers a more comprehensive description of the impact of the disease on patient’s everyday life (Smith et al. 1999). Having a validated instrument for patients with insect venom allergy will allow us for a greater understanding of the specific subjective experiences of venom allergy in our country.

Furthermore, the inclusion of patients living in different geographic regions of the country, confirms that the questionnaire is suitable for the majority of the population. The Turkish version of the VQLQ can therefore be used in advance research as well as in clinical routine in patients with vespid or bee venom allergy to assess somatopsychic stress and anxiety. It is also appropriate for monitoring the influence on QoL of venom immunotherapy.

One of the major limitation of the study is that construct validity has only been tested against the expectation of outcome questions. It would have been useful to examine convergent and divergent validity by looking at correlations with other validated scales.

In summary, here we present that the Turkish version of VQLQ enables the measurement of QoL in patients with vespid as well as bee venom allergy. We could reproduce the sufficient validity and reliability of the original version using the Turkish version of the VQLQ. Furthermore, the responsiveness of this instrument demonstrates the questionnaire’s ability to detect changes over time. Therefore it can be of use for further studies in our patients with insect venom allergy to determine both their psychological status and response to interventions.
